# The dynamic Colombian sign language dataset for basic conversation LSC70

**DOI:** 10.1016/j.dib.2024.111213

**Published:** 2024-12-07

**Authors:** Jader Alejandro Muñoz Galindez, Leidy Verónica Villota Coral, Rubiel Vargas Cañas

**Affiliations:** Sistemas dinámicos, instrumentación y control (SIDICO), Departamento de física, Universidad del Cauca, Colombia

**Keywords:** Alphanumeric, Artificial intelligence, Communication, Deafness, Interpretation, Words

## Abstract

Sign language is a form of non-verbal communication used by people with hearing disability. This form of communication relies on the use of signs, gestures, facial expressions, and more. Considering that in Colombia, the population with hearing impairments is around half a million, a database of dynamic, alphanumeric signs and commonly used words was created to establish a basic conversation. For this purpose, 70 non-expert volunteers in Colombian Sign Language participated, and they were recorded against a clear background under uncontrolled lighting and clothing conditions. The dataset was named LSC70 and includes a six-frame transition in JPG format. It is organized into three parts: The first part contains alphanumeric signs at a resolution of 640×480 pixels; the second part includes word signs at a size of 640×480 pixels; and the third part focuses on the dominant hand in alphanumeric signs at 120×120 pixels. Through filtering that removed similar signs, the dataset now contains 35,208 frames with a total of 47 different signs. These images have been validated by experts at the University of Cauca and are available for free use within the research community.

Specifications Table

**Table:** 

Subject	Computer Vision and Pattern Recognition.
Specific subject area	Sign recognition in people with hearing disability*.*
Type of data	Images in .jpg format separated by participants and categories.
Data collection	This database contains a total of 47 signs in six-frame transitions in JPG format, captured with a Huawei P20 Lite cell phone under uncontrolled lighting and clothing conditions. In total, 35,208 images are collected, including signs for the alphabet, numbers, and common words. The dataset is divided into three sections: LSC70AN, which includes full-body signs of the alphabet and numbers at a resolution of 640×480 pixels; LSC70ANH, which covers alphanumeric signs similarly but focuses on the dominant hand of the volunteers at a resolution of 120×120 pixels; and LSC70 W, which encompasses common words used in basic conversations, such as greetings and identifications, also at 640×480 pixel*.*
Data source location	Institution: Universidad del Cauca, City/Region: Popayán-Cauca, Country: Colombia
Data accessibility	Repository name: Mendeley Data Data identification number: 10.17632/9ssyn8tff5.2 Direct URL to data: https://data.mendeley.com/datasets/9ssyn8tff5/2
Related research article	

## 1 Value of the Data

• A dataset is provided with 35,208 photographs of Colombian language sign performed by 70 people executing 47 signs dynamically under uncontrolled lighting and clothing conditions.
• This dataset allows for the creation and training of artificial intelligence models for interpreting Colombian Sign Language, enabling basic expressions of personal introductions and sharing personal preferences.
• The dataset could be useful for researchers in fields such as technology, linguistics, and sociology, interested in evaluating communication in Colombian Sign Language with the goal of promoting social inclusion.

## 2 Background

Sign language is a non-verbal communication method and the primary means of communication for people with hearing impairments. Globally, the World Health Organization (WHO) estimates that around 5 % of the population (430 million people) suffer from disabling hearing loss and require rehabilitation [1]. In Colombia, estimates from the National Administrative Department of Statistics (DANE) and the National Institute for the Deaf (INSOR) suggest a population of around half a million [2]. Like spoken languages, it has its own vocabulary, idiomatic expressions, and unique grammar. It uses signs, gestures, and facial expressions to effectively communicate ideas, thoughts, and emotions [3].

Colombian Sign Language (CSL) reflects influences from Spanish Sign Language, brought by immigrants or Colombian deaf individuals educated in Spain during the 1950s [4]. This influence results in globally shared signs, such as alphanumeric characters, although there are also variations in special characters, like the letter “ñ.” These differences can vary by region and are influenced by local customs and traditions. Currently, under Law 324 of 1996, CSL is considered part of Colombia's intangible cultural and linguistic heritage, with the national government ensuring its preservation and promotion to guarantee the rights of people with hearing impairments and to foster social inclusion [5,6].

## 3 Data Description

**Fig. 1:**
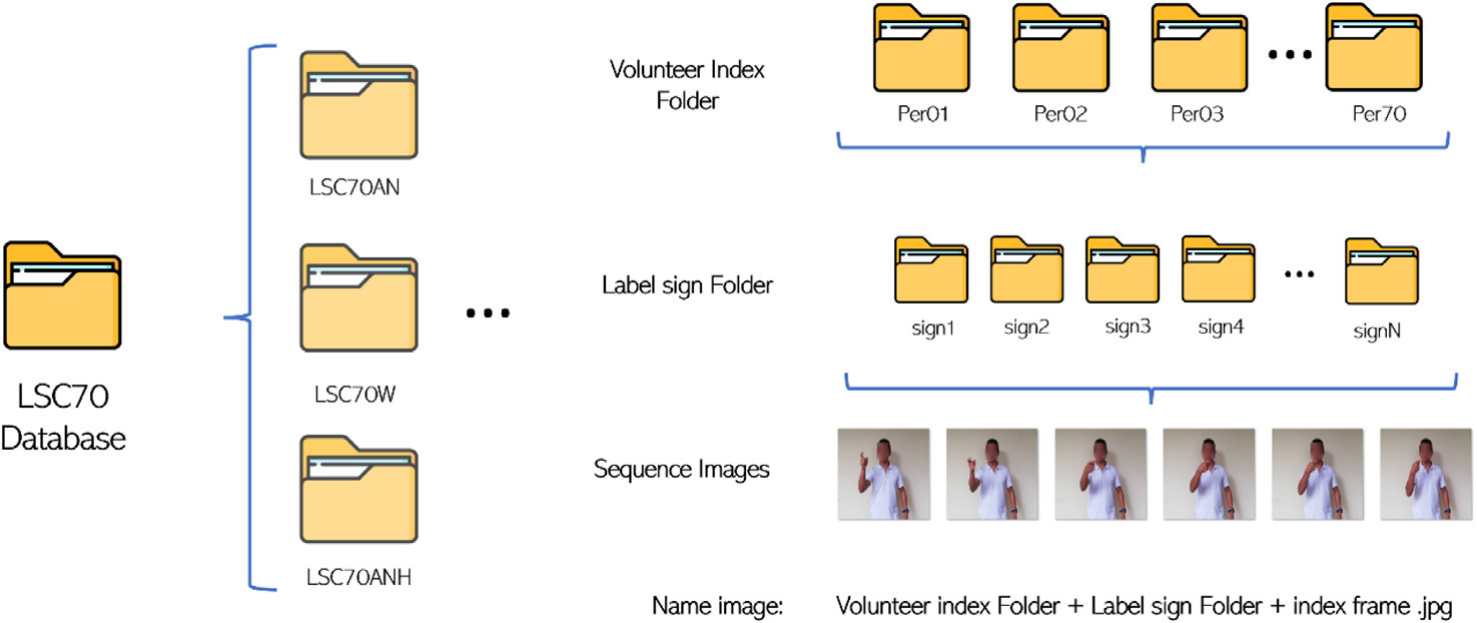
Description of Dataset Storage.

The LSC70 dataset contains a total of 47 signs from CLS, including alphabet, numbers, and common words, in static or dynamic six-frame types, with a total of 35,208 images. The consideration of these signs was based on developing a basic conversation in sign language, involving personal introductions and questions (Table 1), consisting of static signs with no frame-to-frame transition and dynamic signs with a clearly identifiable transition. The dataset is divided into three parts, each with a specific name for recognition. The first part, LSC70AN, focuses on alphanumeric signs; the second, LSC70W, on common words; and finally, LSC70ANH shows the transition of the dominant hand performing alphanumeric signs. This image transition is in “.jpg” format, and the dataset is organized considering the sub-dataset, volunteer index, sign, and finally, the frame number, accompanied by a name that summarizes the destination path such as: “Per + Number + Label + Frame Number.jpg” (Fig. 1).

**Table 1:** Signs Contained in LSC70.

LSC70 database			
Alphabet		Numbers	Words
A	Ñ *	Zero	Hello *
B	O	One	Good *
C	P	Two	Days *
D	Q	Three	Afternoon
E	R	Four	Night *
F	S *	Five	I *
G *	T	Six *	Name *
H *	U	Seven *	Year *
I	V	Eight *	Like *
J *	W	Nine *	Liquor *
K	X	Ten *	
L	Y	Thousand *	
M	Z *	Million *	
N			

*Note: The signs marked with an asterisk * refer to dynamic signs.

### 3.1 LSC70AN

**Fig. 2:**
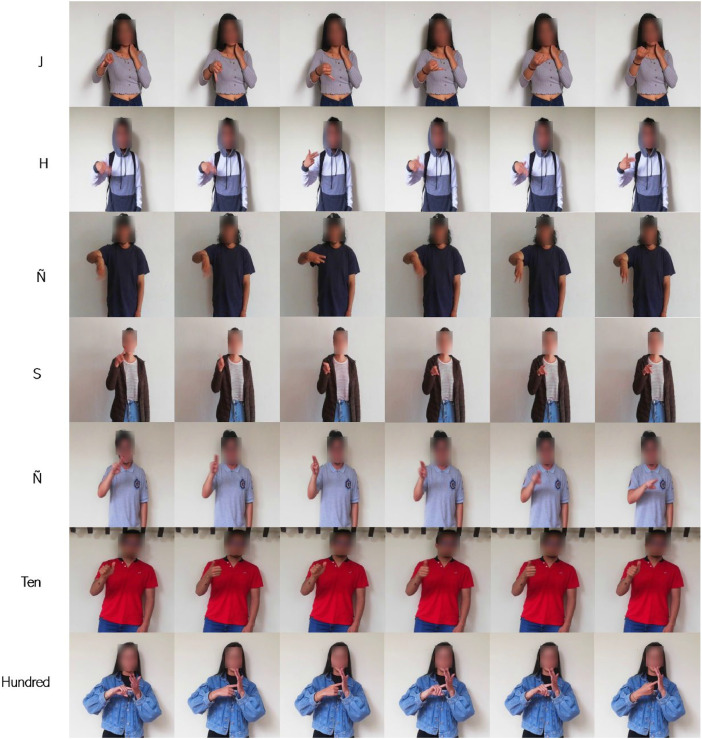
Some dynamic signs present in LSC70AN.

This dataset has a size of 640×480 pixels and includes the 27 letters of the alphabet, as well as 13 signs corresponding to numbers from zero to ten and large quantities to express thousand and million (Fig. 2). Due to the dactylological nature, some similar signs between letters and numbers were omitted to avoid confusion, with the interpretation depending on the context of the conversation. As a result, there is a reduction in the amount of information, decreasing from 40 to 37 signs, which also reduces the number of frames from 16,764 to 15,504. Additionally, this dataset is distributed with 11,340 images of the alphabet and 5424 images of numbers, allowing for communication through spelling.

### 3.2 LSC70ANH

**Fig. 3:**
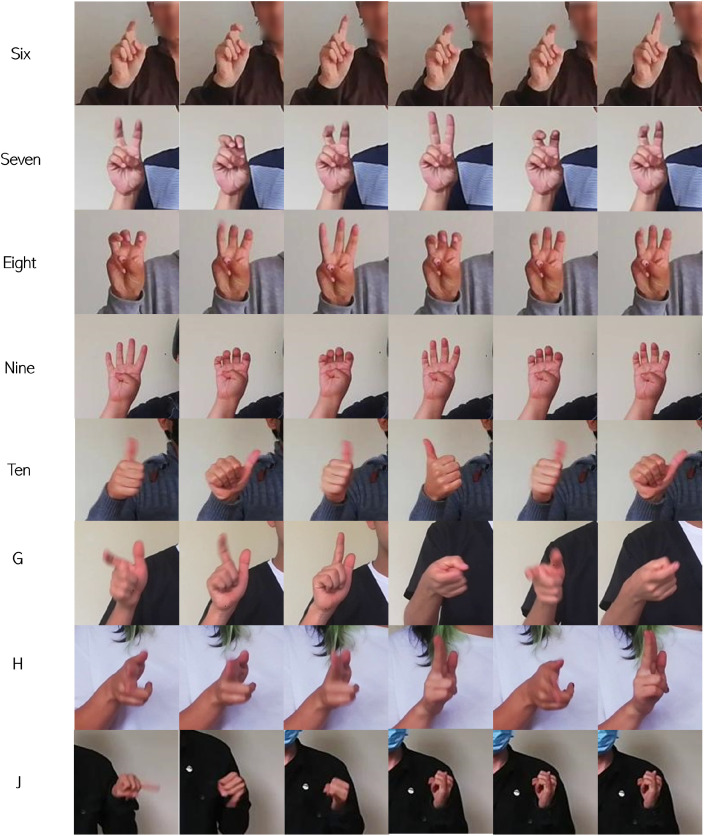
Transition of dynamic signs focused on the hand.

Similar to LSC70AN, this dataset contains the same amount of data and labels. The difference lies in the focus on the dominant hand of the volunteer performing the sign. This process was aimed at detecting the dominant upper limb. The images show the transition and primarily focus on the fingers at a size of 120×120 pixels, allowing for observation of subtle movements during the signs (Fig. 3).

### 3.3 LSC70W

**Fig. 4:**
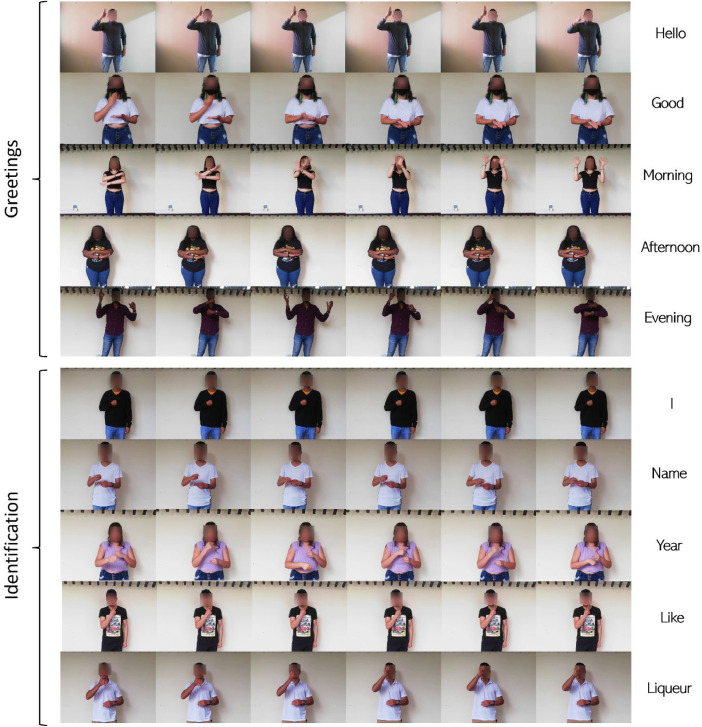
Contents of the signs in LSC70W.

This last dataset comprises the transition of common words used in basic conversation, including greetings such as “Hello,” “Good,” and their derivatives “Days,” “Afternoons,” and “Evening”; and identification terms like “I,” “Name,” “Years,” “Like,” and “Liquor” (Fig. 4). The amount of information is similarly related to the 70 volunteers, resulting in a total of 4200 images at a size of 640×480 pixels. Due to the extension of the arms, these signs are relatively easy to identify; however, they are dependent on the direction of movement, allowing the same sign to have different interpretations.

## 4 Experimental Design, Materials and Methods

### 4.1 Dataset collection

The presented datasets primarily focus on the development of strict communication through the hands. Although facial and body expressions are important and can enrich communication, they are not considered in this context due to the complexity they could add to the communication process. The combination of manual gestures with these additional expressions introduces new nuances and can significantly influence the transmitted meaning.

**Fig. 5:**
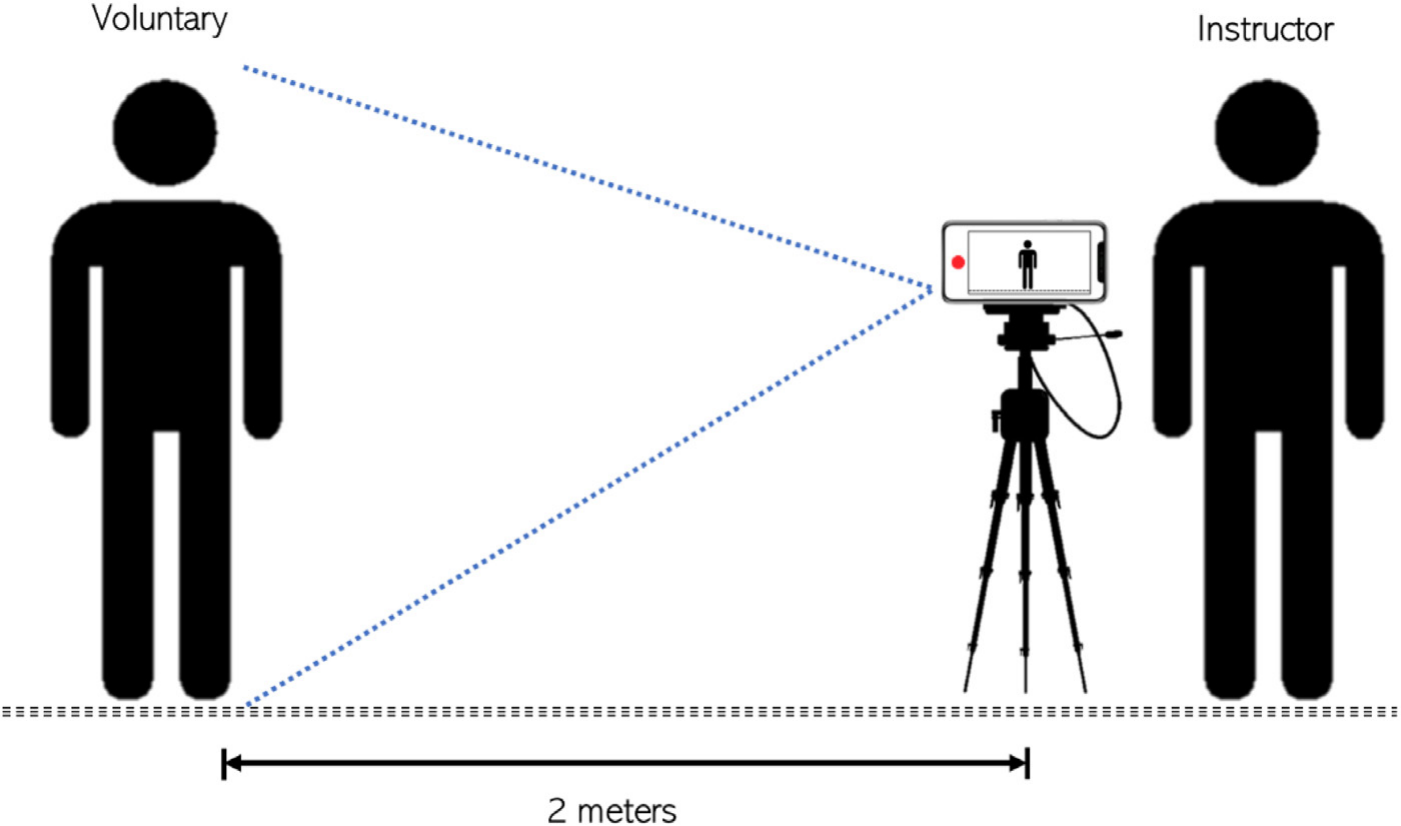
Video acquisition method.

The collection of these data was carried out through video recordings against a clear background under uncontrolled lighting conditions. Seventy-six non-expert volunteers in CLS participated, who provided informed consent to be recorded, and their age and dominant hand data were quantified (Table 2). A 10-min instruction period was given to the participants due to the simplicity of some signs. To provide additional support, the instructor was positioned behind the camera demonstrating how to perform the signs correctly (Fig. 5), while the participant was positioned 2 m in front of the camera. Additionally, to verify the content of the recording, a speech therapist specialized in sign language who works at the institution reviewed the recordings to ensure that the forms aligned with the communication used in Colombia.

**Table 2:** Quantification of participants by sex, age, and dominant hand.

Gender	Quantity	Age range	Right-Handed	Left-Handed
Male	45	18–23	44	1
Female	32	19–29	30	2

Each volunteer was recorded in an individual video performing all the signs presented in Table 1. The recording, which lasted between three and five minutes, included brief pauses between each sign and was done using a Huawei P20 Lite cell phone at a resolution of 640×480 pixels and a refresh rate of 30fps. It was decided to record in low resolution to enable artificial intelligence models to better generalize information, as processing in low resolution is more challenging compared to high resolution. Additionally, many neural network architectures, such as convolutional networks, perform resizing to lower resolutions. Therefore, recording in high resolution was not considered, as it would ultimately be used in low resolution.

The recording started with the alphabet, followed by numbers, and finally, words. Each sign was performed correctly once, and if there was any difficulty, different repetitions were made within the same recording. On average, it took each participant about three minutes to complete all the signs. Once the capture process was finished, the collected data was reviewed and information regarding the size and duration of the videos was extracted. The data was organized by default name and backup copies were made both locally and digitally to prevent data loss.

**Fig. 6:**
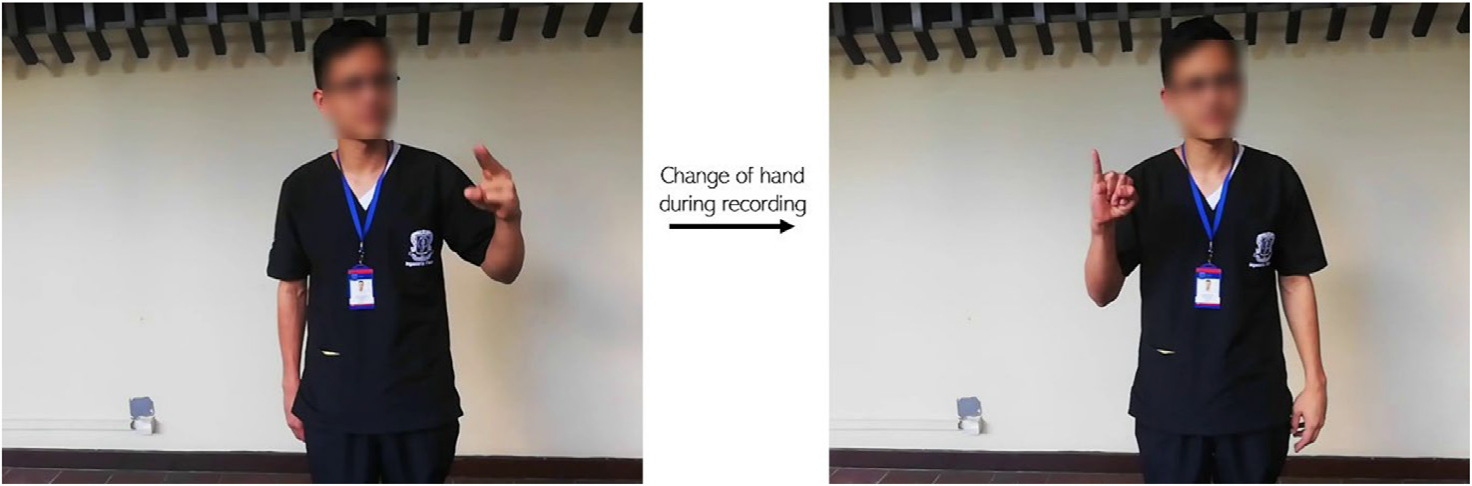
Hand change during recording due to muscle fatigue.

Control over clothing was not considered, which allowed some colors or patterns to potentially be visual distractions or affect the clarity of the signs. Additionally, the most common clothing items or accessories worn by the volunteers were quantified to identify any potential obstructions (Table 3), considering environmental conditions on the recording day and the health of the participants. The most common items were coats and face masks due to the rainy season during the recording. On the other hand, the major issue observed among participants was muscle fatigue while performing the signs, which led to changing the hand executing the sign shortly after the recording began or confusion regarding the dominant limb itself (Fig. 6).

**Table 3:** Quantification of clothing and accessories.

Caps	Face Masks	Coats	Glasses
4	14	23	18

### 4.2 Data preparation

To understand the peculiarities and difficulties that may have arisen during the sign recording process, each recording was reviewed individually, and information was filtered based on parameters to determine a usable recording (Table 4). In total, six videos were omitted where some participants had significant difficulty performing the signs. This results in 70 videos that meet the appropriate characteristics for use.

**Table 4:** Parameters for considering videos as usable.

Number of Repetitions	Background Visibility	Hand Change	Accesories
3	Yes	Yes	Yes

Once the videos were selected, frames were extracted using an algorithm developed in Python with the open-source library OpenCV. The sampling frequency was 6 fps, maintaining a resolution of 640×480 in JPG format, with extraction intervals set at 166 ms. These six frames were extracted, considering the execution of the signs within the first second. No additional processing was applied to enhance the lighting conditions or the quality of the images, and there was no need to include metadata.

The storage was automatically organized, creating a folder for each volunteer, with subfolders arranged by each sign, providing an index order for the extracted frames. Additionally, a general filtering was performed for similar signs, specifically for the numbers zero, two, and three, which correspond to the consonants O, V, and W respectively (Table 5). This filtering aimed to reduce repeated signs that do not contribute different or relevant frames.

**Table 5:** Similarity between consonant signs and numbers.

Letter	Sign	Sign	Number
O			Zero
V			Two
W			Three

**Fig. 7:**
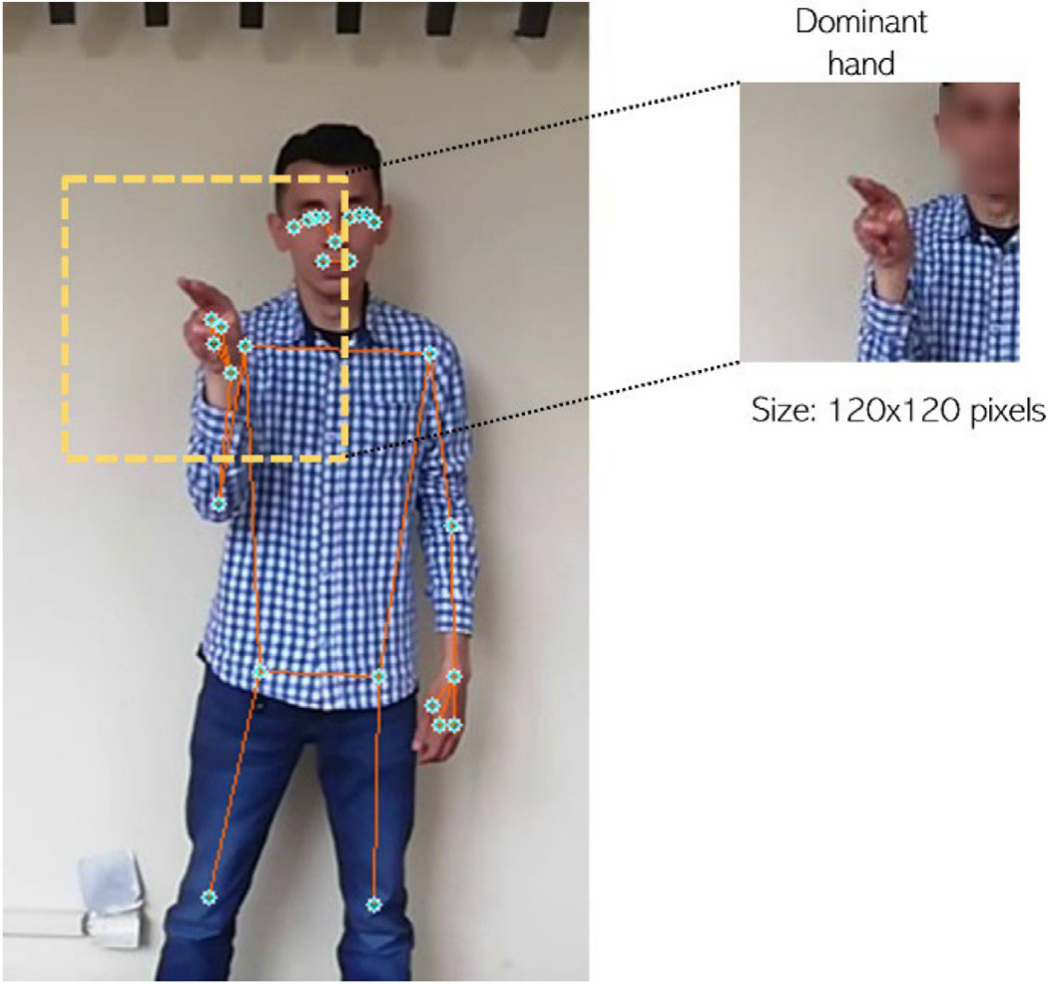
Focus on dominant hand using MediaPipe.

Additionally, to increase the amount of data on alphanumeric signs, MediaPipe Pose is used, focusing on points 20 or 19 (Fig. 7), corresponding to the dominant right or left hand for upper limb detection. The reason for creating this second repository focused on the dominant hand was to compare it with similar repositories found in the literature, which mainly focus on the hand in a static manner [2,7]. These typically address-controlled conditions where the background is easily removed. In this case, the approach used does not maintain a relationship with the background, as the signs can be transferred quickly from one environment to another, making it very similar to normal conditions of communication in sign language. On the other hand, considering a resolution of 120×120 pixels does not significantly affect the ability to distinguish the signs, as other repositories, such as CIFAR-10 [8] and MNIST [9], use a low resolution without this implying deficient results in their modeling, as it ultimately depends on the architecture of the artificial intelligence model.

## Limitations

The low resolution of the recordings can cause some signs to appear blurred and not show the transition clearly between frames. Additionally, since the participants are not experts in CLS, it is noticeable that they have difficulty performing some signs correctly, and the time taken to perform them can extend beyond one second, which may result in incomplete capture of the transitions.

## Ethics Statement

During the development of the recordings, certain ethical guidelines were adopted, including obtaining written informed consent. This was provided after explaining the objective of the recording, the possible uses of the data collected and the rights of the participants. Data protection and privacy were guaranteed, avoiding the disclosure of any personal information and keeping identification confidential. Participation was completely voluntary, with the possibility of withdrawing from the recording at any time. All necessary preventive measures were implemented to minimize any discomfort or risk during recording. The data collected will be used exclusively for scientific research purposes, always preserving respect for the dignity, rights and well-being of the participants. Because the identity of the participants was protected and their informed consent was obtained, institutional ethics committee approval was not required, although all procedures were carried out in strict observance of ethical standards for research involving human subjects.

## CRediT Author Statement

**Jader Muñoz**: Data curation, Conceptualization, Methodology, Software, Writing – original draft, Writing – review & editing; **Leidy Villota**: Data curation, Software, Writing - original draft, Writing – review & editing; **Rubiel Vargas**: Conceptualization, Methodology, Supervision, Writing – original draft.

## Data Availability

Mendeley DataSpeak in Colombian Sign Language: A Dynamic LSC70 Database (Original data).
